# An Unusual Presentation of Severe Hypothyroidism in a Child With Undiagnosed Systemic Lupus Erythematous

**DOI:** 10.1210/jcemcr/luaf309

**Published:** 2025-12-30

**Authors:** Josephine Elliott, Eman Kamaleldeen, Penelope Davis, Renuka P Dias, Kristien Boelaert, Zainaba Mohamed

**Affiliations:** Institute of Cancer and Genomic Sciences, University of Birmingham, Birmingham B15 2TT, UK; Department of Paediatric Endocrinology and Diabetes, Birmingham Women's and Children's Hospital NHS Foundation Trust, Birmingham B4 6NH, UK; Department of Paediatric Rheumatology, Birmingham Women's and Children's Hospital NHS Foundation Trust, Birmingham B4 6NH, UK; Department of Paediatric Endocrinology and Diabetes, Birmingham Women's and Children's Hospital NHS Foundation Trust, Birmingham B4 6NH, UK; Department of Applied Health, School of Health Sciences, College of Medicine and Health, University of Birmingham, Birmingham B15 2TT, UK; Department of Applied Health, School of Health Sciences, College of Medicine and Health, University of Birmingham, Birmingham B15 2TT, UK; Department of Paediatric Endocrinology and Diabetes, Birmingham Women's and Children's Hospital NHS Foundation Trust, Birmingham B4 6NH, UK

**Keywords:** thyroid, autoimmune, levothyroxine

## Abstract

Myxedema is increasingly rare in the United Kingdom, particularly among patients already on treatment. We present a case of a 13-year-old girl diagnosed with autoimmune hypothyroidism who was started on 75 µg of levothyroxine by her general practitioner. She was referred to the endocrine clinic after 6 months of treatment for hypothyroidism with 2 months of worsening fatigue, chest pain, peripheral edema, and dyspnea.

On hospitalization, the patient’s laboratory tests revealed undetectable free thyroxine level (fT4) and elevated thyrotropin (TSH) greater than 500 μIU/mL (SI: >500 m mIU/L) (reference range, 0.51-4.30 μIU/mL [SI: 0.51-4.30 mIU/L]). She was diagnosed with thyroid myxedema and commenced intravenous (IV) hydrocortisone, IV maintenance fluids, and antibiotics.

However, the constellation of signs and symptoms remained perplexing. Further tests revealed a large pericardial effusion, deep vein thrombosis, and nephritis. A multidisciplinary approach confirmed the diagnosis of systemic lupus erythematous (SLE), and the patient was successfully started on rituximab therapy within 5 days of admission.

This case illustrates the importance of considering potential underlying diagnoses when assessing patients with hypothyroid crisis, especially when symptoms deviate from the typical clinical presentation. The timely recognition and management of this patient's underlying SLE were crucial in her recovery.

## Introduction

Hypothyroidism affects up to 5% of the general population and is diagnosed biochemically with elevated thyrotropin (TSH) and low free thyroxine (fT4) levels [1]. Symptoms include weight gain, constipation, fatigue, depression, and goiter [2]. Myxedema is a rare, life-threatening complication of hypothyroidism with an estimated incidence of 0.22 per 1 000 000 per year in Western countries, typically resulting from prolonged untreated hypothyroidism [3]. We describe a patient with rapid-onset myxedema despite treatment compliance with levothyroxine (L-T4).

## Case Presentation

A previously well 13-year-old girl was referred to pediatric endocrinology in January 2025 for autoimmune hypothyroidism, diagnosed by her general practitioner (GP) in July 2024 after presenting with fatigue and muscle weakness. Blood tests showed elevated TSH at 40.6 μIU/mL (SI: 40.6 mIU/L) (reference range [RR], 0.51-4.30 μIU/mL [SI: 0.51-4.30 mIU/L]) and positive thyroid peroxidase (TPO) antibodies at 35 kU/L (SI: 35 IU/mL) (RR, 0-5.6 kU/L [SI: 0-5.6 IU/mL]). Her GP started her on 75 µg L-T4 once daily.

Soon after, the patient experienced hair loss, which she attributed to the medication. The dosage was halved for 2 weeks, then returned to the full dose of 75 µg by the GP. She remained compliant, taking it consistently before breakfast. Despite treatment, she continued to have excessive fatigue, bilateral leg swelling, and severe muscle weakness, significantly affecting her daily activities and resulting in more than a month of school absence. Additionally, she reported cold intolerance and considerable weight loss. Follow-up thyroid function tests in December 2024 with her GP showed TSH of 24.7 μIU/mL (SI: 24.7 mIU/L) and fT4 of 13.6 pmol/L (SI: 1.06 ng/dL) (RR, 11.3-20.3 pmol/L [SI: 0.88-1.58 ng/dL]).

In the 4 weeks before endocrine clinic review, she developed chest pain, palpitations, and dyspnea. She had no significant medical or family history.

On examination in the endocrine clinic, the patient had very dry skin, cool peripheries, hyperpigmentation of her knuckles, alopecia, a displaced apex heartbeat to the seventh intercostal space, muffled heart sounds, proximal weakness of the upper arms, delayed knee jerk reflexes, striae on her knees and abdomen, and painless, pitting edema to the mid-shin. Her right leg was more swollen than the left, but both calves were soft and nontender. She had a smooth grade 2 goiter [4], with no bruits and no nodules, that did not extend retrosternally. She had a malar rash that appeared intermittently. She was tachycardic at 139 beats per minutes (bpm), with a blood pressure of 119/80 mm Hg, temperature of 38.6 °C, and capillary blood glucose of 4.4 mmol/L.

She was immediately referred to the emergency department for admission in view of hemodynamic instability. She was short of breath, unable to speak in full sentences, and unable to walk unaided. Observations on admission showed a heart rate of 140 bpm, respiratory rate of 22 breaths per minute, temperature of 37.7 °C, oxygen saturations of 99% in air, and blood pressure of 115/74 mm Hg. She had appropriate auxology for her age with a weight on the 25th percentile and a height on the 50th percentile.

## Diagnostic Assessment

The patient’s laboratory work-up on hospital admission showed severe hypothyroidism with an fT4 of 6.8 pmol/L (SI: 0.53 ng/dL), TSH of 519.40 μIU/mL (SI: 519.40 mIU/L), and elevated TPO antibodies at 54.7 kU/L (SI: 54.7 IU/mL), confirming severe hypothyroidism. She passed an urgent 60-minute short synacthen test (with 250 μg of synacthen), achieving a cortisol peak of 69 μg/dL (SI: 690 nmol/L) (RR, >50 μg/dL [SI: >500 μg/L]).

Hematology results showed a microcytic anemia with a hemoglobin of 7.1 g/dL (SI: 71 g/L) (RR, 12-16 g/dL [SI: 120–160 g/L]) and a normal clotting screen.

Renal test results showed a stage 1 acute kidney injury with a urine protein:creatinine ratio of 8.43 mg/mg (SI: 952.4 mg/mmol) (RR, <0.18 mg/mg [SI: <20 mg/mmol]). Serum albumin was low at 21 g/L (SI RR, 43-54 g/L).

**Figure 1.**
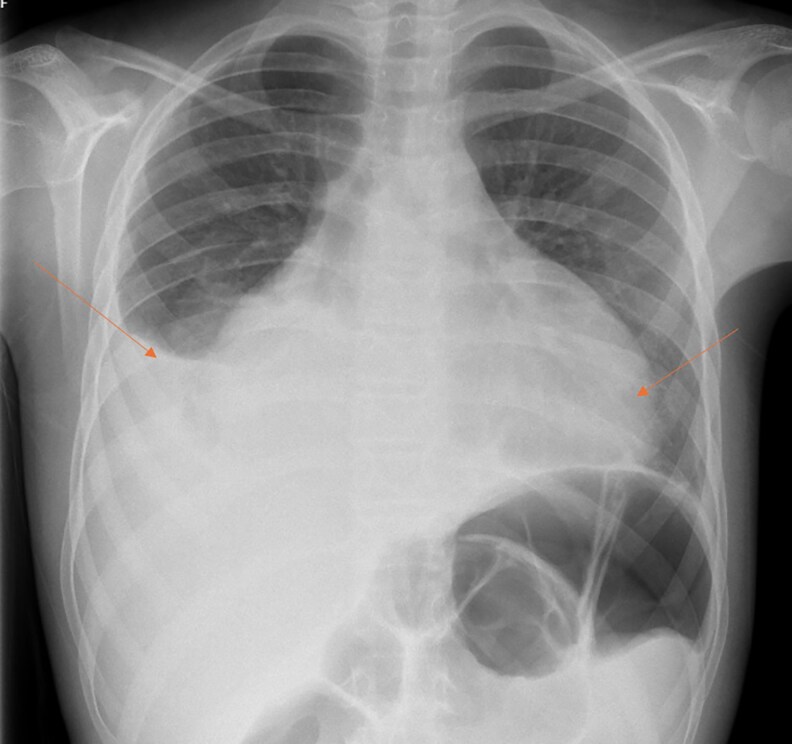
Chest radiograph taken the day of admission, depicting cardiomegaly and large right pleural effusion.

Cardiac investigations demonstrated an elevated N-terminal pro-B-type natriuretic peptide (BNP) at 2398 ng/L (SI: 2398 pg/mL) (RR, 0-400 ng/L [SI: 0–400 pg/mL]), suggesting right-sided heart strain myocarditis. Electrocardiogram showed sinus rhythm with left axis deviation. Her chest radiograph on admission showed cardiomegaly and a large right-sided pleural effusion with a meniscus (Fig. 1). Ultrasound of the thorax showed a moderate right anechoic pleural effusion with consolidation of the right lower lobe of the lung, a small left pleural effusion, and a large pericardial effusion. An echocardiogram showed mild mitral regurgitation and a medium-large circumferential pericardial effusion.

On day 2 of admission, during insertion of an ultrasound-guided cannula, a right upper-limb thrombus was incidentally discovered. An urgent Doppler of her right arm and leg revealed a right occlusive ileofemoral deep vein thrombus (DVT). There were no features of pulmonary embolus on computed tomography (CT) pulmonary angiogram scan.

In view of these additional findings, the differential diagnosis was broadened. Her microcytic anemia, prothrombotic state, and weight loss caused concern for an underlying malignancy; however, a CT scan of the chest, abdomen, and pelvis showed no features of this. The constellation of malar rash, renal involvement, pleural effusion, and prothrombotic state led to seeking a rheumatology opinion for systemic lupus erythematous (SLE) on day 3 of admission.

In the second week of admission, the patient’s blood tests indicated raised double-stranded DNA antibodies (>379.0 IU/mL [SI RR, 0.0-10.0 IU/mL]) and antinuclear antibodies (>1:1280). She had low complement C3 at 0.30 g/L (SI RR, 0.75-1.65 g/L) and low complement C4 at 4 mg/dL (SI: 0.04 g/L) (RR, 14-54 g/L [SI: 0.14-0.54 g/L]). These results, along with the clinical presentation, confirmed the diagnosis of SLE. Anticardiolipin and lupus anticoagulant antibodies were negative.

## Treatment

**Figure 2.**
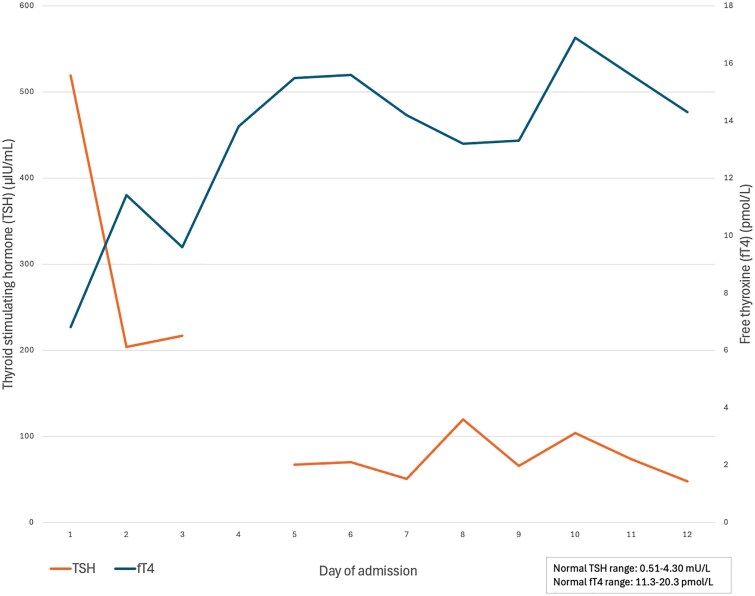
Graphical representation of thyroid function test trends in the first 12 days of treatment, showing a sharp drop in thyrotropin levels following administration of intravenous levothyroxine and a slow, steady rise in fT4 levels to within normal range. fT4, free levothyroxine hormone; TSH, thyrotropin.

After her short synacthen test on admission, the patient received IV hydrocortisone (100 mg), followed by stress dosing (30 mg/m²/day in 4 divided doses). On day 2 of admission, she was initiated on IV 200 μg L-T4 once daily and oral 100 μg L-T4 once daily, along with IV maintenance fluids (0.9% sodium chloride with 5% dextrose). By the following day, her fatigue and shortness of breath had significantly improved. IV L-T4 was discontinued after 3 days, and oral L-T4 was continued and titrated as her thyroid function improved (Fig. 2).

In view of her pleural effusion and concerns for a chest infection, she was started on IV amoxicillin with clavulanic acid for 3 days, but this was stopped when blood cultures were confirmed negative and inflammatory markers, except for erythrocyte sedimentation rate, remained low. Enoxaparin was started for her DVT, then bridged to warfarin.

On day 4 of admission, she commenced first-line therapy for presumed SLE with methylprednisolone, hydroxychloroquine, and lansoprazole for gastroprotection. In light of her lupus nephritis, she was commenced on rituximab.

## Outcome and Follow-up

The patient made an excellent recovery and was discharged from hospital after 1 month. Her pleural and cardiac effusions resolved, her fT4 normalized within 4 days of treatment (see Fig. 2), and her TSH normalized within 3 months of initial admission. The patient is followed up in rheumatology, endocrine, and hematology clinics, with close monitoring of her thyroid function tests. Four months following hospital discharge, she reports feeling well, with good energy levels, and her leg swelling continues to slowly resolve.

## Discussion

We describe a young girl with rapid decompensation of hypothyroidism into life-threatening myxedema in the context of untreated SLE. There has been one other case report of a pediatric patient presenting with myxedema and underlying SLE [5]. However, to the best of our knowledge, this is the first recorded case of myxedema occurring despite the patient already taking thyroid replacement treatment, and we think it important to consider why this happened.

SLE increases the risk of developing other autoimmune diseases, including hypothyroidism [6]. Our patient's SLE likely contributed to the rapid onset of myxedema through several mechanisms. First, patients with SLE and hypothyroidism are at risk of more pronounced disease manifestation and increased risk of organ damage [7]. Second, the nephrotic syndrome secondary to SLE probably played a part in preventing the gut absorption of the oral L-T4 that our patient had been taking during the 6 months prior to hospital admission. Gut malabsorption in SLE can lead to loss of thyroid-binding globulins, albumin, and transition proteins, causing a reduction in the circulating thyroid hormone levels [8]. Third, inflammatory cytokines can suppress the hypothalamic-pituitary-thyroid axis, alter thyroid hormone–binding proteins, and reduce peripheral conversion of T4 to 3,5,3′-triiodothyronine (T3), leading to reduced fT4 and T3 concentrations [9-11]. Thus, the coexistence of autoimmune hypothyroidism and SLE may compound the biochemical abnormalities. In these ways, the primary pathology of SLE decompensated an otherwise stable patient with hypothyroidism, despite her treatment compliance, causing life-threatening myxedema.

We chose to treat her myxedema with L-T4 rather than T3 in the initial phase because T3 has more potent effects on metabolism, especially in the heart [12], causing concern for arrhythmias and high-output cardiac failure in a patient who already had a significant pericardial effusion and tachycardia. The American Thyroid Association guidelines for hypothyroidism treatment also recommend IV L-T4 [13].

We treated with steroids because chronic hypothyroidism may suppress the hypothalamic-pituitary-adrenal axis, potentially leading to adrenal insufficiency. This mechanism has been suggested both in animal and clinical studies, although direct evidence in humans remains limited [14-16]. The short synacthen test is commonly used to assess adrenal function; however, its diagnostic accuracy may be affected in acutely ill patients due to technical and interpretative factors [17]. Our patient's short synacthen test showed a cortisol peak of only 69 μg/dL (SI: 690 μg/L) at admission, despite the immense stress her body was under, suggesting that adrenal insufficiency could not be ruled out. If there had been an undiagnosed adrenal insufficiency, the IV L-T4 treatment alone could have precipitated adrenal crisis [18]. This is due to replacement thyroid hormones increasing metabolic demand with the risk of unmasking or worsening adrenal insufficiency by increasing cortisol clearance.

Patients with SLE are at significantly increased risk of thromboembolic events [19]. Our patient's DVT was likely multifactorial, owing to her muscle weakness reducing her mobility, her nephrotic syndrome predisposing her to thrombus formation [20], and her hypothyroidism causing an increased coagulopathic state secondary to the effect of thyroid hormones on the synthesis of hemostatic factors [21]. Considering this risk, it would be appropriate for involved clinicians to screen for DVT early if there is leg swelling.

In conclusion, this is a case of a young girl who presented with rapid-onset myxedema despite treatment of hypothyroidism due to an underlying diagnosis of SLE. We highlight the complexity of diagnosing and treating myxedema with multiorgan damage due to a second underlying autoimmune condition that was untreated. The timely recognition and management of this patient's underlying autoimmune condition was pivotal in her recovery.

## Learning Points

- Clinicians should be alert for potential underlying autoimmune conditions when assessing patients with rapid-onset hypothyroid crisis.
- Nephrotic syndrome secondary to SLE can lead to reduced absorption of oral L-T4; even a patient on treatment for hypothyroidism for months can present in myxedema crisis.
- SLE and myxedema can increase the risk of DVT even without associated antiphospholipid syndrome.

## Contributors

All authors made individual contributions to authorship. All authors were involved in the diagnosis and management of this patient. Z.M., R.P.D., and P.D. were consultants directly responsible for the patient's care; J.E. and E.K. were involved in the patient's inpatient care, and K.B. advised on patient management. J.E. wrote the case report and all coauthors gave feedback and approved the final version of the report. The patient and her family also reviewed and approved the case report.

## Data Availability

Original data generated and analyzed during this study are included in this published article.

## Abbreviations

bpm: beats per minutes
CT: computed tomography
DVT: deep vein thrombosis
fT4: free thyroxine
GP: general practitioner
IV: intravenous
L-T4: levothyroxine
RR: reference range
SLE: systemic lupus erythematosus
T3: 3,5,3′-triiodothyronine
TPO: thyroid peroxidase
TSH: thyrotropin
